# Comparison of *matK* and *rbcL* DNA barcodes for genetic classification of jewel orchid accessions in Vietnam

**DOI:** 10.1186/s43141-021-00188-1

**Published:** 2021-06-21

**Authors:** Viet The Ho, Thi Kim Phuong Tran, Thi Thanh Tram Vu, Sasanti Widiarsih

**Affiliations:** 1 Ho Chi Minh City University of Food Industry, 140 Le Trong Tan, Tan Phu District, Ho Chi Minh City, Vietnam; 2 Plant Mutation Breeding Division, Centre for Isotopes and Radiation Application, National Nuclear Energy Agency, Jl. Lebakbulus Raya No. 49, South Jakarta, Indonesia

**Keywords:** DNA barcode, Genetic diversity, Jewel orchid, *matK*, *rbcL*

## Abstract

**Background:**

Jewel orchid is the common name of several orchid species which can be alike in morphological characteristics, but variable in medicinal properties. At present, two DNA barcode loci, namely, *maturase K* (*matK*) and *ribulose 1,5-biphosphate carboxylase* (*rbcL*), are intensively utilized for plant identification. However, the discrimination effectiveness of these loci is variable among plant species. This study was carried out to compare the identifying efficacy of these two loci on jewel orchid population collected throughout Vietnam.

**Results:**

The results revealed that 21 jewel orchid accessions studied were segregated into four different species with significant variations. The discrimination power of *matK* and *rbcL* markers in this jewel orchid study displayed different efficiency level. The *rbcL* gene has higher distinguishing potential than either *matK* gene alone or the combination of both genes.

**Conclusion:**

The findings of this project could provide valuable information that is necessary for classification, plant origin identification, breeding, and conservation program of jewel orchid in Vietnam.

**Supplementary Information:**

The online version contains supplementary material available at 10.1186/s43141-021-00188-1.

## Background

The term “jewel orchid” refers to several species of orchid of velvety brocade-like leaves with beautiful veins. They belong to a diverse plant group of Orchidaceae family which spread widely in tropical regions of Asia and Australia, and have high medicinal and economic values. As traditional medicine, jewel orchid is used to treat chest and abdominal pain, diabetes, nephritis, fever, hypertension, liver, and pleurisy. Several chemical compounds have been identified by advanced analytics methods to show strong biological activity which can improve the lung and liver conditions [1].

Several jewel orchid species may share nearly similar morphological characteristics although their economic and pharmaceutical values are very different. Therefore, an accurate classification of this orchid group of high medicinal properties as a basis for development and conservation is urgently needed. However, the current plant identification is still using the traditional classification method: rely on the morphological characteristics of leaves, flowers, and stems. There are some problems encountered from applying this method, such as nearly identical external morphology features, variable polymorphisms between adult and juvenile stages, and environmental factors as well as the plant growth development phases; all leads to inaccuracy. Also, morphological identification cannot be performed properly if the specimen has been damaged or has been subjected to preliminary processing. Applying the incorrect species with different pharmaceutical compounds as herbal medicine would reduce the effectiveness of the medicine, and could be harmful to the patients.

Recently, DNA barcode is increasingly becoming a more popular method to identify species, utilizing reliable DNA regions. It is used worldwide to serve the classification, biodiversity assessment, and genetic resource conservation, and also to overcome the limitation of morphology-based taxonomy. As a relatively new technique, DNA barcode uses the standardized genomic regions to distinguish among species and has been used intensively for identifying at species level. In animals, the mitochondrial cytochrome oxidase I (COI) gene was generally used for phylogenetic study. However; the same gene cannot be employed in plants, as it lacks sufficient variations due to low mutation rate [2]. For plants, other gene regions have been utilized as DNA barcodes, such as nuclear ribosomal internal transcribed space (ITS) [3, 4], also *rbcL*, *matK*, *atpF-atpH*, *psbK-psbI*, and *trnH-psbA* [5–7].

In Orchidaceae family, DNA barcode has been used intensively to species identification or classification. Kim and colleagues developed DNA barcodes for 89 orchid species in Korea [5]. A study using *rbcL*, *matK*, ITS, and *trnH-psbA* barcodes was also effective for identification of endangered orchid in *Paphiopedilum* species in Malaysia [7]. In Vietnam, Huynh and colleagues employed up to nine DNA barcodes to discover the species diversity of six jewel orchid accessions [8]. A large study in China has screened 1698 accessions of 184 *Dendrobium* species with 11 candidate barcodes, and then proposed that due to the easiness in amplification and sequencing, the primer sets suitable for *Dendrobium* orchid study were ITS, ITS2, *matK*, *rbcL*, and *trnH-pbsA* [6].

Among several barcode loci, *matK* and *rbcL* were proposed as the preferred plant barcoding loci by The Consortium for the Barcode of Life (CBOL) [9]. Nevertheless, the ideal locus for DNA barcoding of plants remains debatable, since some loci are efficient for some specific taxonomic groups only and the species discrimination of these genes varies among plant species. When studied *Aquilaria* genus, Thitikornpong and colleagues discovered more variation in *matK* gene in comparison to *rbcL* gene [10]; similar result has also been found in phylogenetic analysis of *Dalbergia* [11]. A variation of species resolution in different vascular plant species was exhibited by both *rbcL* and *matK* [12], whereas *rbcL* has better performance in teak, black rosewook, ben teak [13], and also liverwort [14]. Therefore, the purpose of this study was to evaluate the species resolution ability of *matK* and *rbcL* loci in 21 accessions jewel orchid collected in Vietnam. The obtained results will be useful for genetic conservation and breeding purposes. Furthermore, the markers that are found to be tightly linked to specific accessions will also pave the way for classification, conservation, and protection of this plant group.

## Methods

**Fig. 1:**
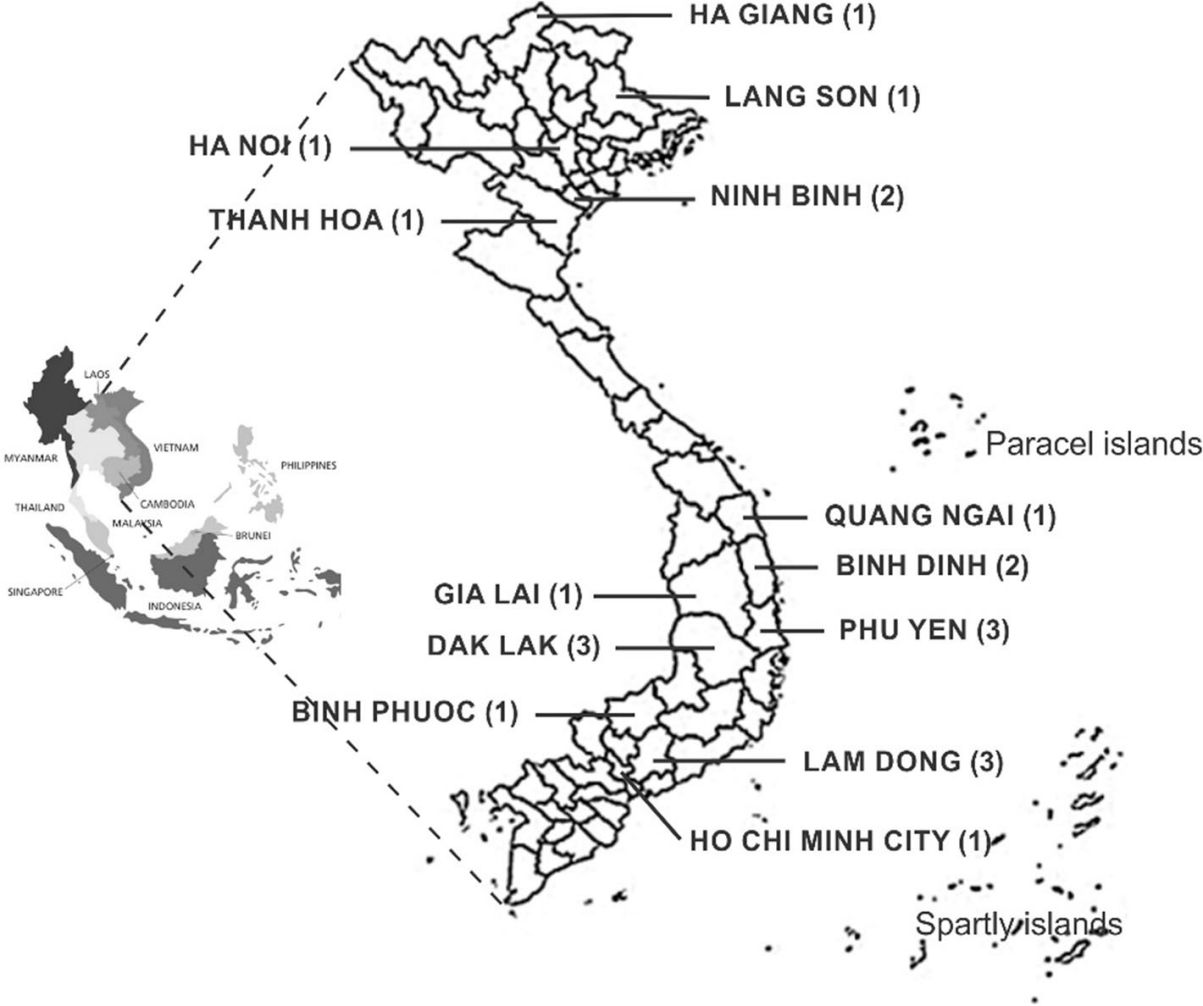
Targeted areas for collecting jewel accessions in this study (sample numbers collected in each location are indicated in parentheses)

A total of 21 jewel orchid accessions were collected from different places in Vietnam (Fig. 1 and Table 1). The leaf samples were dried in silica gel and stored at room temperature until usage.

**Table 1:** Jewel orchid samples collected for genetic characterization and the corresponding accession numbers

No.	Sample code	Collection site	Accession number for ***matK*** gene	Accession number for ***rbcL*** gene
1	HG	Ha Giang Province	MW241553	MW241574
2	LS	Lang Son Province	MW241554	MW241575
3	HN	Ha Noi City	MW241555	MW241576
4	NB1	Ninh Binh Province	MW241556	MW241577
5	NB2	Ninh Binh Province	MW241557	MW241578
6	TH	Thanh Hoa Province	MW241558	MW241579
7	QN	Quang Ngai Province	MW241559	MW241580
8	BD1	Binh Dinh Province	MW241560	MW241581
9	BD2	Binh Dinh Province	MW241561	MW241582
10	GL	Gia Lai Province	MW241562	MW241583
11	PY1	Phu Yen Province	MW241563	MW241584
12	PY2	Phu Yen Province	MW241564	MW241585
13	PY3	Phu Yen Province	MW241565	MW241586
14	DL1	Dak Lak Province	MW241566	MW241587
15	DL2	Dak Lak Province	MW241567	MW241588
16	DL3	Dak Lak Province	MW241568	MW241589
17	BP	Binh Phuoc Province	MW241569	MW241590
18	LD1	Lam Dong Province	MW241570	MW241591
19	LD2	Lam Dong Province	MW241571	MW241592
20	LD3	Lam Dong Province	MW241572	MW241593
21	HCM	Ho Chi Minh City	MW241573	MW241594

DNA was extracted with CTAB method (cetyl trimethyl ammonium bromide) as described by Doyle and Doyle [15]. PCR reaction for *matK* and *rbcL* regions was amplified using the composition as follows: 7.5 μL 2X Mytaq Mix (Bioline, UK), 20 ng DNA, 0.2 μM primer (either *matK* 390F: 5′-CGATCTATTCATTCAATATTTC-3′; and 1326R: 5′-TCTAGCACACGAAAGTCGAAGT-3′ [16] or *rbcL*: cF: 5′-TGAAAACGTGAATTCCCAACCGTTTATGCG-3′; cR: 5′-GCAGCAGCTAGTTCCGGGCTCCA-3′ [17], and PCR water (Sigma-Aldrich, USA) to final volume of 15 μL. The PCR reaction conditions were as follows: initial denaturation at 95 °C for 2 min; then 35 cycles of 30 s at 95 °C, 30 s at 55 °C, and 1 min at 72 °C. Finally, an additional of 5 min was continued at 72 °C to complete the reaction. All reactions were carried out in SureCycler 8800 Thermal Cycler (Agilent, USA). The PCR products were electrophorized on 1% agarose gel using 1 kb DNA marker (Bioline, UK) to confirm the amplification length. The PCR products were then purified by ISOLATE II PCR and Gel Kit (Bioline, UK) and sequenced using the BigDyeTM Terminator Cycle Sequencing Kit (Applied Biosystem, USA). The products were next run on ABI 3100 DNA analyzer (Applied Biosystem, USA). The obtained electropherograms were edited using FinchTV (Digital World Biology Products, USA). Only the sequences with scores higher than 20 PHRED score were considered for further analysis. Sequences were trimmed at both ends of the alignment in order to avoid too many missing data at the ends. The obtained sequences were submitted to GenBank (NCBI, USA) and are publicly accessible under the accession numbers listed in Table 1.

For species identification, the DNA sequences were identified with Barcode of Life Database (BOLD) system in the function of *rbcL* and *matK* for plants. Similarly, the homology of *matK* and *rbcL* sequences was checked simultaneously with Basic Local Alignment Search Tools (BLAST) of NCBI using default parameters. The identification was deemed correct if the highest identity percentage of searched sequences was derived from expected species or genus. On the other hand, the identification was considered ambiguous when the highest identity percentage of searched sequences was not derived from expected species or genus or family [18]. DNA sequences were then aligned with the ClustalW algorithm, implemented in MEGA7 package [19], using the default parameters. Evolutionary divergence for each data set and pattern of nucleotide substitution were performed on the same software. Evolutionary trees were constructed based on two methods: maximum likelihood (ML) and neighbor joining (NJ), each represents for discrete character methods and distance methods, respectively [20]. The reliability of phylogenetic analysis was validated by 1000 bootstrap replicates. Bootstrap support (BS) was categorized as strong (> 85%), moderate (70-85%), weak (50-69%), or poor (< 50%) [21].

In order to estimate species resolution for a given barcode locus, we considered the species were resolved if conspecific individual grouped into one monophyletic branch in the phylogenetic tree with strong bootstrap support. On the other hand, if conspecific individuals were separated in paraphyletic branches, then it was considered as identification failure [22]. The correlation between the *matK* and *rbcL* similarity matrices were computed by Mantel test at a significant level of 5% in 1000 simulations by using program Mantel test of Microsoft Excel 2010 [23].

## Results

### Species identification

In this study, both *matK* and *rbcL* sequences were successfully sequenced. For homologous identification, only sequences of minimum 80% percentage identity were considered. Using BLAST, both *matK* and *rbcL* genes were showing identical results as described in Table 2.

**Table 2:** Searching result of *matK* and *rbcL* gen on Genbank and BOLD databases

No.	Sample code	BLAST with ***matK***	BOLD with ***matK***	BLAST with ***rbcL***	BOLD with ***rbcL***
1	HG	*Goodyera schlechtendaliana*	*Goodyera oblongifolia*	*Goodyera schlechtendaliana*	*Platythelys querceticola*
2	LS	*Ludisia discolor*	*Ludisia discolor*	*Ludisia discolor*	*Platythelys querceticola*
3	HN	*Goodyera velutina*	*Ludisia discolor*	*Goodyera velutina*	*Cephalanthera falcata forma*
4	NB1	*Ludisia discolor*	*Ludisia discolor*	*Ludisia discolor*	*Platythelys querceticola*
5	NB2	*Ludisia discolor*	*Platylepis polyadenia*	*Ludisia discolor*	*Platythelys querceticola*
6	TH	*Goodyera velutina*	*Anoectochilus formosanus*	*Goodyera velutina*	*Cephalanthera falcata forma*
7	QN	*Ludisia discolor*	*Zeuxine nervosa*	*Ludisia discolor*	*Platythelys querceticola*
8	BD1	*Anoectochilus pingbianensis*	*Ludisia discolor*	*Anoectochilus pingbianensis*	*Platythelys querceticola*
9	BD2	*Ludisia discolor*	*Anoectochilus roxburghii*	*Ludisia discolor*	*Platythelys querceticola*
10	GL	*Goodyera velutina*	*Zeuxine nervosa*	*Goodyera velutina*	*Cephalanthera falcata forma*
11	PY1	*Ludisia discolor*	*Ludisia discolor*	*Ludisia discolor*	*Platythelys querceticola*
12	PY2	*Anoectochilus roxburghii*	*Anoectochilus formosanus*	*Anoectochilus roxburghii*	*Nothoalsomitra suberosa*
13	PY3	*Ludisia discolor*	*Zeuxine nervosa*	*Ludisia discolor*	*Platythelys querceticola*
14	DL1	*Ludisia discolor*	*Ludisia discolor*	*Ludisia discolor*	*Platythelys querceticola*
15	DL2	*Ludisia discolor*	*Zeuxine nervosa*	*Ludisia discolor*	*Platythelys querceticola*
16	DL3	*Ludisia discolor*	*Ludisia discolor*	*Ludisia discolor*	*Platythelys querceticola*
17	BP	*Anoectochilus pingbianensis*	*Goodyera pubescens*	*Anoectochilus pingbianensis*	*Platythelys querceticola*
18	LD1	*Anoectochilus roxburghii*	*Anoectochilus formosanus*	*Anoectochilus roxburghii*	*Nothoalsomitra suberosa*
19	LD2	*Anoectochilus pingbianensis*	*Zeuxine nervosa*	*Anoectochilus pingbianensis*	*Platythelys querceticola*
20	LD3	*Anoectochilus pingbianensis*	*Anoectochilus roxburghii*	*Anoectochilus pingbianensis*	*Platythelys querceticola*
21	HCM	*Anoectochilus roxburghii*	*Anoectochilus roxburghii*	*Anoectochilus roxburghii*	*Cucumis sativus*

Using BLAST for searching homology, the results of *matK* and *rbcL* are identical. On the contrary, the results from BOLD were totally different and the returned species from this database were not corresponding to those of BLAST. Furthermore, the obtained results from *matK* and *rbcL* by BOLD were also not consistent. *MatK* sequences show higher similarity to that of BLAST with 7/21 accessions with identical results. Nevertheless, none of returned results from *rbcL* was identical to that of BLAST. Even more, several returned identifications were completely irrelevant to jewel orchid. Limited accessions were shown as belonged to other genus in Orchidaceae family, such as *Platythelys querceticola or Cephalanthera falcata* forma. In particular, LD1 and HCM accessions were shown as belonged to two genuses in Cucurbitaceae family: *Nothoalsomitra suberosa* and *Cucumis sativus*, respectively.

### Estimation of sequence divergence

The divergence among sequences is slightly variable (Supplementary table S1). Among which, the divergence value of *matK* and *rbcL* regions was ranged from 0 to 0.14 and from 0 to 0.05, respectively. In *matK* region, PY2 accession showed a higher difference from those of other species, which vary from 0.09 to 0.14; whereas *rbcL* from HG accession showed the highest divergence, which vary from 0.0 to 0.05. The substitution of different bases in analyzed regions was evaluated on entire codon positions (1st+ 2nd + 3rd nucleotide) and was displayed in Table 3. In general, the transitional substitution is higher than the transversional substitution in both *matK* and *rbcL* regions. However, *matK* region exhibited a higher substitution rate from G to A. In contrast, the changing frequency from C to T, T to C, and A to G of *rbcL* was higher than that of *matK*.

**Table 3:** Pattern of nucleotide substitution of *matK* and *rbcL* regions (in percentage)

	***matK***				***rbcL***			
	A	T	C	G	A	T	C	G
A	-	9.27	4.07	**8.36**	-	6.06	3.82	**12.87**
T	7.43	-	**7.67**	3.44	5.68	-	**12.84**	5.59
C	7.34	**17.45**	-	3.44	5.68	**20.36**	-	5.59
G	**18.10**	9.27	4.07	-	**12.36**	6.06	3.82	-

Note: Substitution pattern and rates were estimated under the Tamura-Nei (1993) model. Rates of different transitional substitutions are shown in bold and those of transversional substitutions are shown in italics

Furthermore, two parameters were utilized to examine the inter-specific divergence; consisted of average inter-specific distance and range of inter-specific distance. Another two parameters, namely, average intra-specific distance and range of intra-specific distance, were used to evaluate the intra-specific divergence. The obtained results revealed that *matK* possessed the higher intraspecific distance and lower interspecific distance (Table 4).

**Table 4:** Estimates of average evolutionary divergence of *matK* and *rbcL* sequences

Parameters	*matK*	*rbcL*
Range of intraspecific distance	0.01-0.04	0-0.01
Range of interspecific distance	0.0219-0.0496	0.003-0.112
Intraspecific distance (mean)	0.025	0.0025
Interspecific distance (mean)	0.034	0.051

### Phylogenetic analyses

**Fig. 2:**
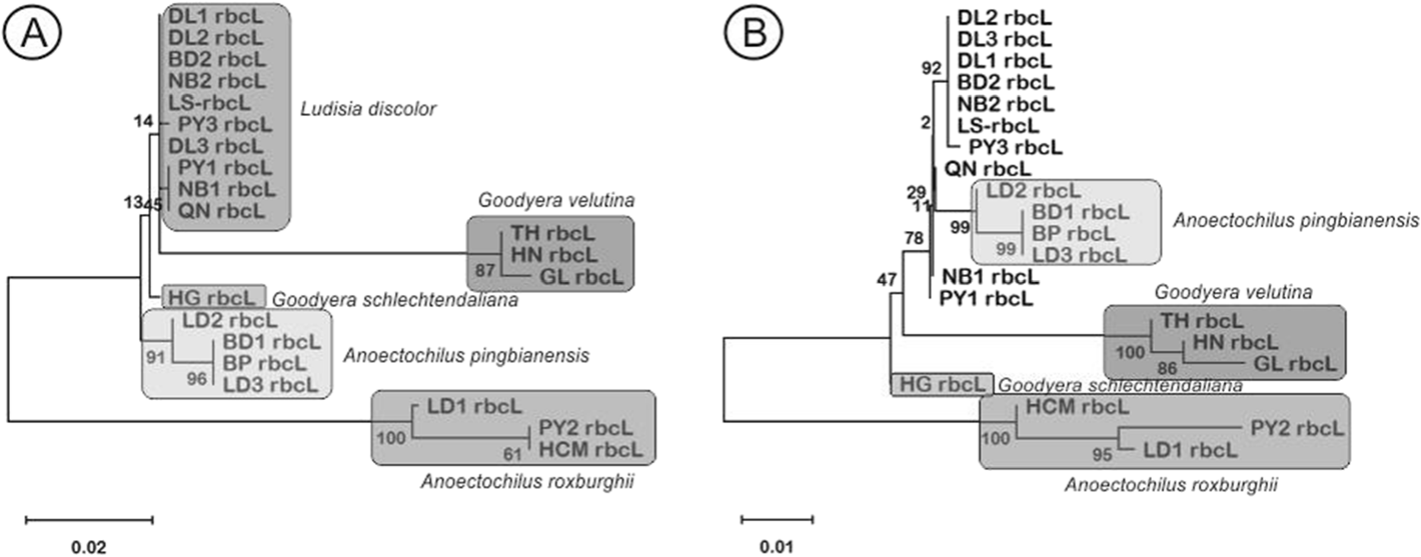
Phylogenetic tree based on *rbcL* region of 21 jewel orchid accessions by maximum likelihood (**A**) and neighbor joining (**B**). The value in horizontal bar explains the length of the branch, which represents the number of nucleotide substitution

By employing ML, 21 accessions were successfully classified into five separate groups, which were also corresponding to the five species identified by BLAST (Table 2). However, when utilizing NJ, only four species: *Anoetochilius pingbianeisis*, *Goodyere velunitna*, *Goodyera schlechtendalinana*, and *Anoectochilus roxburghii,* were correctly grouped, while the remaining accessions belonging to *Ludisia discolor* were divided into two subgroups (Fig. 2).

**Fig. 3:**
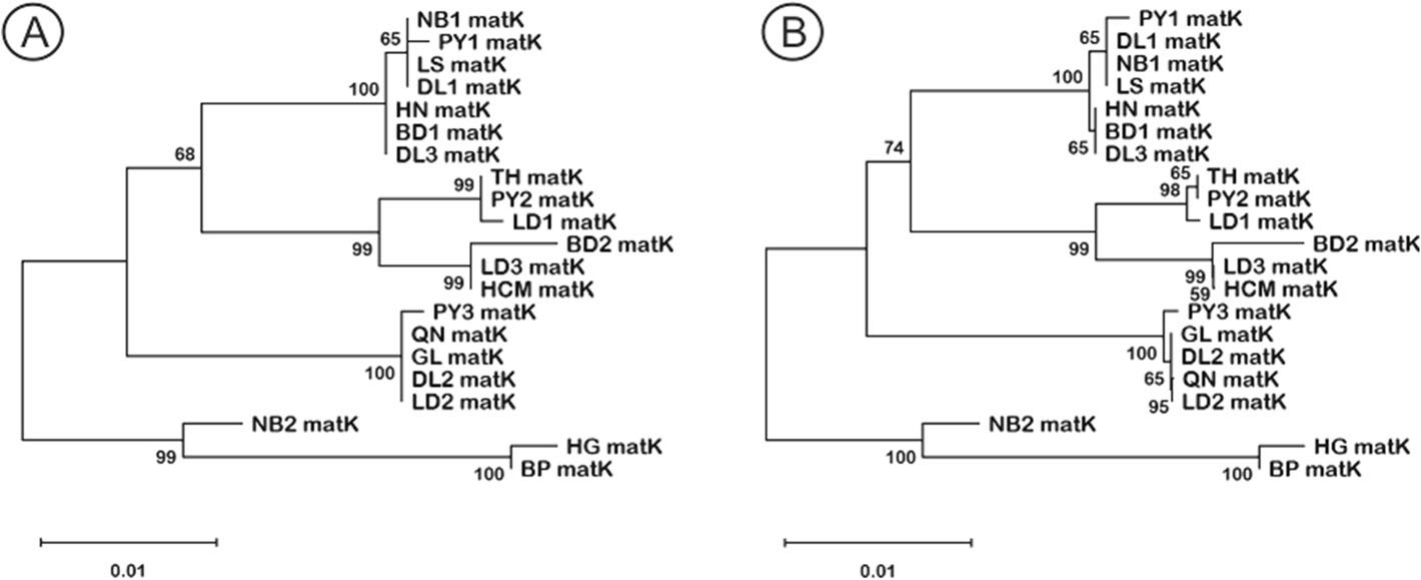
Phylogenetic tree based on *matK* region of 21 jewel orchid accessions by maximum likelihood (**A**) and neighbor joining (**B**). The value in horizontal bar explains the length of the branch, which representing the number of nucleotide substitution

In contrast to *rbcL* region, phylogenetic analysis of *matK* was failed to show any clear grouping for both ML and NJ analysis (Fig. 3).

**Fig. 4:**
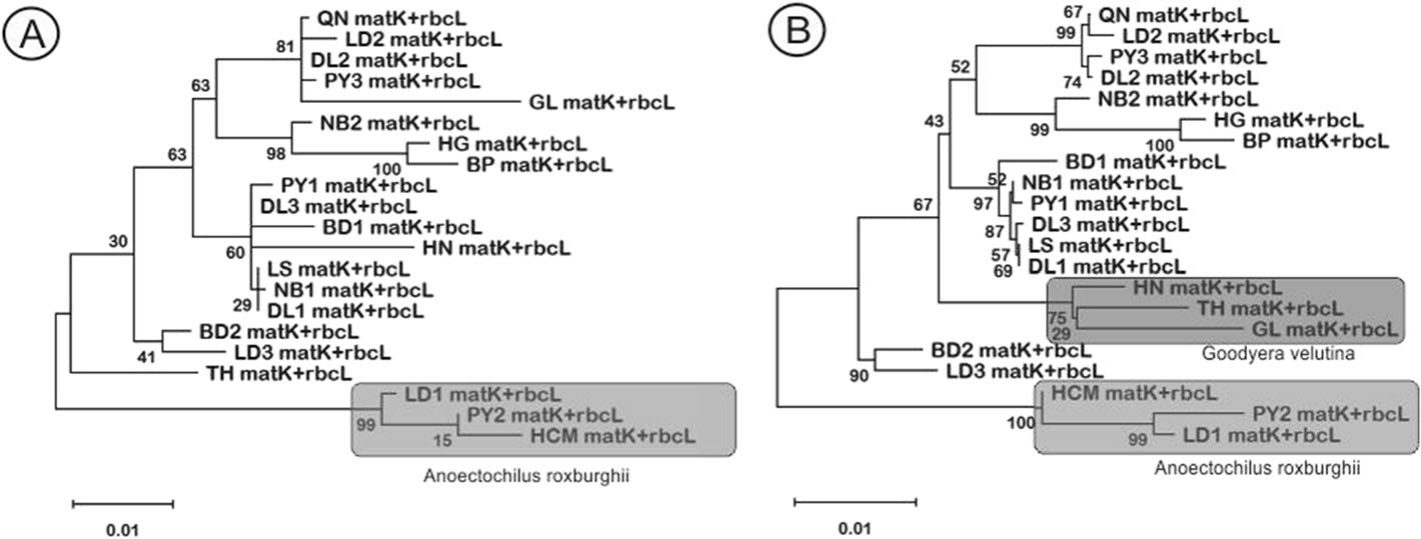
Phylogenetic tree based on the combination of *matK* and *rbcL* sequences of 21 jewel orchid accessions by maximum likelihood (**A**) and neighbor joining (**B**)

The combination of two barcode regions was unsuccessful to increase the species power resolution compared to the single use. The phylogenetic tree was resulted in one and two separate branches when using ML and NJ methods, respectively (Fig. 4). Mantel’s test also failed to find the relatedness between *matK* and *rbcL* barcodes with P value = 0.883.

## Discussion

### Species identification

Although numerous studies mentioned the low sequencing effectiveness of *matK* region in vascular plants in the comparison to *rbcL* [24], no problem was encountered on our DNA sequencing of both genes. The BLAST results were consistent in searching for homology of both *matK* and *rbcL* genes. On the other hand, the BOLD results were totally different. The low accuracy of BOLD could be originated from the small size and insufficient completeness of their database. Consequently, the missing species in the database cannot be identified and the method may assign the query sequence to an incorrect species [25]. Similar result was previously reported on *Chenopodium murale* [26]. Their study reported that when using BLAST, the specimen was identified as *Chenopodium murale* for both *matK* and *rbcL* gene with 100% sequence similarity. Whereas when using BOLD, *rbcL* gene showed high similarity, ranged from 96.3 to 100% with different species such as *C. ambrosiodies*, *C. album*, and *C. ficifolium*.

### Estimation of sequence divergence

In molecular evolution study, estimation of nucleotide substitution is vital to show the presence of genetic divergence. In our study, the divergence value of *matK* and *rbcL* regions ranged from 0 to 0.14 and 0 to 0.05, respectively. This value is significantly lower than previous data reported by Sikdar and colleagues when analyzing 46 *rbcL* sequences and 42 *matK* sequences of 21 species in Fabaceae family [22]. Higher divergence of *matK* marker has been widely reported and made *matK* being considered as highly potential barcoding regions for systematic and evolution study in plants [27]. For example, *matK* was proved to be more divergent than *rbcL* at both intra-specificity and inter-specificity in a study of the *rbcL* and *matK* region effectiveness for 490 vascular plant species [12].

Two parameters in this study were utilized to examine the inter-specific divergence: average inter-specific distance and range of inter-specific distance. Another two parameters were used to evaluate intra-specific divergence, namely, average intra-specific distance, and range of intra-specific distance. In general, a desirable barcode gene should have high inter-specific divergence and low intra-specific divergence; thus, *rbcL* is superior to *matK* in this jewel orchid study. A study on several medical plants also revealed that *rbcL* has lowest intra-specific distance in the comparison to other common barcode regions in plant study such as ITS, ITS2, *psbA-trnH*, ycf5, and *rpoC1* [28]. Ideally, the interspecific distance value of DNA barcode should be higher than that of intraspecific distance to produce non-overlapping value or “barcoding gap,” which in turn will increase the discrimination power of barcode in classification study. However, the barcode gap is absent in this study, suggesting that the studied species are closely related.

### Phylogenetic analyses

The resolution capacity of a barcode is its ability to differentiate and identify species based on interspecific differences among DNA sequences. A species is considered as resolved if its individuals construct a specific monophyletic branch. The result shows that ML is more effective in species classification of jewel orchid. Although both ML and NJ are commonly used in phylogenetic analysis, NJ can be easily performed in a short time with personal computer while ML is considered as professional method in phylogenetic analysis. ML could consider the possibility for all events happening simultaneously and produced the best tree, supported at higher probability in comparison to other methods [29]. In which the homologous variations from alignment results will be focused. This method has been used to identify several plants such as *Epimedium elatum* [30].

The evolution of *matK* region is considered as the fastest in plastid genome and the sequence is highly similar to COI sequence in animal which is commonly used as key barcode region in animal identification. However, in contrast to *rbcL* region, phylogenetic analysis of *matK* did not show any clear group for both ML and NJ analysis. This is also supported by Mantel’s test. Numerous studies have been reported superiority of *rbcL* in plant classification such as in Palmae family [24]; *Codiaeum varieagatum* [18]; and Ranunculaceae family [31]. Similarly, when Maloukh and colleagues studied the discriminatory power for authentication purpose of DNA barcode on 51 plant species in United Arab Emirates, *rbcL* successfully identified 100% (51/51) plant species including 11 monocots and 40 eudicots plant, whereas *matK* resulted in only 24.45% (14/51) of correct species identification [32]. Different DNA barcode markers could affect the resulted phylogenetic tree. A study in Dipterocarpaceae family has shown inconsistency of the phylogenetic tree built by *rbcL* and *matK* genes [33]. Another study on Casuarinaceae found that *matK* gene gave higher resolution than *rbcL* [34]. A research group in Vietnam also reported that *matK* region was a more reliable marker than *rbcL* on *Hopea chinesis* [35].

The combination of multi loci barcodes could improve the species classification [9] and several studies have proven this idea [35–37]. In our study, however, the combination of two barcode region failed to increase the species differentiation power compared to the single one. Previous researches on different trees also reported this phenomenon. For example, a 2019 study on Ranunculanceae family in China showed that the combination of *matK* and *rbcL* showed lower species resolution in contrast to *rbcL* alone for both ML and NJ analysis [31].

## Conclusion

Both *matK* and *rbcL* barcode loci could be used as a complementary tool for jewel orchid identification; however, the effectiveness of each locus should be examined adequately case by case. The combination of two barcode regions was not better than the single one. The results suggest that the discrimination of *rbcL* locus is superior to *matK* locus. Future studies combined with additional barcode loci are necessary to develop a better and more effective differentiation method among different species of jewel orchid.

## Supplementary Information

**Additional file 1: Supplementary table S1**. Estimates of Evolutionary Divergence between DNA barcode Sequences. (The number of base substitutions per site from between sequences of *matK* and *rbcL* regions is shown below and above the diagonal, respectively.

## Data Availability

The datasets used and/or analyzed during the current study are available from the corresponding author on reasonable request.

## Abbreviations

ITS: Internal transcribed space
COI: Cytochrome oxidase I
*matK*: Maturase K
*rbcL*: Ribulose 1,5-biphosphate carboxylase
DNA: Deoxyribonucleic acid
CTAB: Cetyl trimethyl ammonium bromide
BOLD: Barcode of Life Database
BLAST: Basic Local Alignment Search Tools
ML: Maximum likelihood
NJ: Neighbor joining
