# Supplementary material for: Comparison of matK and rbcL DNA barcodes for genetic classification of jewel orchid accessions in Vietnam
Source: J Genet Eng Biotechnol. 2021 Jun 21;19:93. doi: 10.1186/s43141-021-00188-1 (PMC8217478; doi:10.1186/s43141-021-00188-1)
Supplement: Supplementary file 1 — Additional file 1: Supplementary table S1. Estimates of Evolutionary Divergence between DNA barcode Sequences. (The number of base substitutions per site from between sequences of matK and rbcL regions is shown below and above the diagonal, respectively. [file 43141_2021_188_MOESM1_ESM.docx]

Supplementary table S1: Estimates of Evolutionary Divergence between DNA barcode Sequences

|  | **HG** | **LS** | **HN** | **NB1** | **NB2** | **TH** | **QN** | **BD1** | **BD2** | **GL** | **PY1** | **PY2** | **PY3** | **DL1** | **DL2** | **DL3** | **BP** | **LD1** | **LD2** | **LD3** | **HCM** |
| --- | --- | --- | --- | --- | --- | --- | --- | --- | --- | --- | --- | --- | --- | --- | --- | --- | --- | --- | --- | --- | --- |
| **HG** |  | 0.05 | 0.05 | 0.05 | 0.02 | 0.05 | 0.05 | 0.05 | 0.05 | 0.05 | 0.05 | 0.05 | 0.05 | 0.05 | 0.05 | 0.05 | 0.00 | 0.05 | 0.05 | 0.05 | 0.05 |
| **LS** | 0.00 |  | 0.00 | 0.00 | 0.03 | 0.03 | 0.03 | 0.00 | 0.03 | 0.03 | 0.00 | 0.03 | 0.03 | 0.00 | 0.03 | 0.00 | 0.04 | 0.02 | 0.03 | 0.03 | 0.03 |
| **HN** | 0.03 | 0.04 |  | 0.00 | 0.03 | 0.02 | 0.03 | 0.00 | 0.03 | 0.03 | 0.00 | 0.02 | 0.03 | 0.00 | 0.03 | 0.00 | 0.04 | 0.03 | 0.03 | 0.03 | 0.03 |
| **NB1** | 0.00 | 0.00 | 0.04 |  | 0.03 | 0.03 | 0.03 | 0.00 | 0.03 | 0.03 | 0.00 | 0.03 | 0.03 | 0.00 | 0.03 | 0.00 | 0.04 | 0.02 | 0.03 | 0.03 | 0.03 |
| **NB2** | 0.00 | 0.00 | 0.04 | 0.00 |  | 0.04 | 0.03 | 0.03 | 0.04 | 0.03 | 0.03 | 0.04 | 0.03 | 0.03 | 0.03 | 0.03 | 0.02 | 0.04 | 0.03 | 0.03 | 0.04 |
| **TH** | 0.03 | 0.04 | 0.00 | 0.04 | 0.04 |  | 0.04 | 0.02 | 0.02 | 0.04 | 0.03 | 0.00 | 0.04 | 0.03 | 0.04 | 0.02 | 0.05 | 0.00 | 0.04 | 0.01 | 0.01 |
| **QN** | 0.00 | 0.00 | 0.04 | 0.00 | 0.00 | 0.04 |  | 0.03 | 0.04 | 0.00 | 0.03 | 0.04 | 0.00 | 0.03 | 0.00 | 0.03 | 0.05 | 0.04 | 0.00 | 0.03 | 0.04 |
| **BD1** | 0.01 | 0.01 | 0.05 | 0.01 | 0.01 | 0.05 | 0.01 |  | 0.03 | 0.03 | 0.00 | 0.02 | 0.03 | 0.00 | 0.03 | 0.00 | 0.04 | 0.03 | 0.03 | 0.03 | 0.03 |
| **BD2** | 0.00 | 0.00 | 0.04 | 0.00 | 0.00 | 0.04 | 0.00 | 0.01 |  | 0.04 | 0.03 | 0.02 | 0.04 | 0.03 | 0.04 | 0.03 | 0.05 | 0.02 | 0.04 | 0.00 | 0.00 |
| **GL** | 0.05 | 0.05 | 0.00 | 0.05 | 0.05 | 0.00 | 0.05 | 0.07 | 0.05 |  | 0.03 | 0.04 | 0.00 | 0.03 | 0.00 | 0.03 | 0.05 | 0.04 | 0.00 | 0.03 | 0.03 |
| **PY1** | 0.00 | 0.00 | 0.04 | 0.00 | 0.00 | 0.04 | 0.00 | 0.01 | 0.00 | 0.05 |  | 0.03 | 0.03 | 0.00 | 0.03 | 0.00 | 0.05 | 0.03 | 0.03 | 0.03 | 0.03 |
| **PY2** | 0.09 | 0.11 | 0.13 | 0.10 | 0.11 | 0.13 | 0.10 | 0.12 | 0.11 | 0.14 | 0.10 |  | 0.04 | 0.03 | 0.04 | 0.02 | 0.05 | 0.00 | 0.04 | 0.01 | 0.01 |
| **PY3** | 0.01 | 0.00 | 0.04 | 0.00 | 0.00 | 0.04 | 0.00 | 0.02 | 0.00 | 0.06 | 0.00 | 0.11 |  | 0.03 | 0.00 | 0.03 | 0.05 | 0.04 | 0.00 | 0.04 | 0.04 |
| **DL1** | 0.00 | 0.00 | 0.04 | 0.00 | 0.00 | 0.04 | 0.00 | 0.01 | 0.00 | 0.05 | 0.00 | 0.11 | 0.00 |  | 0.03 | 0.00 | 0.04 | 0.02 | 0.03 | 0.03 | 0.03 |
| **DL2** | 0.00 | 0.00 | 0.04 | 0.00 | 0.00 | 0.04 | 0.00 | 0.01 | 0.00 | 0.05 | 0.00 | 0.11 | 0.00 | 0.00 |  | 0.03 | 0.05 | 0.04 | 0.00 | 0.03 | 0.03 |
| **DL3** | 0.00 | 0.00 | 0.04 | 0.00 | 0.00 | 0.04 | 0.00 | 0.01 | 0.00 | 0.05 | 0.00 | 0.11 | 0.00 | 0.00 | 0.00 |  | 0.04 | 0.03 | 0.03 | 0.03 | 0.03 |
| **BP** | 0.01 | 0.01 | 0.05 | 0.01 | 0.01 | 0.05 | 0.01 | 0.00 | 0.01 | 0.07 | 0.01 | 0.12 | 0.02 | 0.01 | 0.01 | 0.01 |  | 0.05 | 0.05 | 0.05 | 0.05 |
| **LD1** | 0.07 | 0.09 | 0.12 | 0.08 | 0.09 | 0.12 | 0.08 | 0.10 | 0.09 | 0.13 | 0.08 | 0.02 | 0.09 | 0.09 | 0.09 | 0.09 | 0.10 |  | 0.04 | 0.01 | 0.01 |
| **LD2** | 0.01 | 0.01 | 0.05 | 0.01 | 0.01 | 0.05 | 0.01 | 0.01 | 0.01 | 0.06 | 0.01 | 0.11 | 0.01 | 0.01 | 0.01 | 0.01 | 0.01 | 0.09 |  | 0.03 | 0.03 |
| **LD3** | 0.01 | 0.01 | 0.05 | 0.01 | 0.01 | 0.05 | 0.01 | 0.00 | 0.01 | 0.07 | 0.01 | 0.12 | 0.02 | 0.01 | 0.01 | 0.01 | 0.00 | 0.10 | 0.01 |  | 0.00 |
| **HCM** | 0.05 | 0.05 | 0.07 | 0.05 | 0.05 | 0.07 | 0.05 | 0.06 | 0.05 | 0.09 | 0.05 | 0.00 | 0.05 | 0.05 | 0.05 | 0.05 | 0.06 | 0.01 | 0.05 | 0.06 |  |

(The number of base substitutions per site from between sequences of *matK* and *rbcL* regions is shown below and above the diagonal, respectively.
